# The role of notch signaling pathway and non-coding RNAs in cancer and inflammation: progress, therapeutic insights, and future directions

**DOI:** 10.3389/fimmu.2025.1567040

**Published:** 2025-06-20

**Authors:** Rong-Zu Nie, Hang Wang, Shuang-Shuang Wang, Chen Chen, Huo-Min Luo, Hao-Kun Zhang, Zhao-Hui Jing, Pei-Feng Li

**Affiliations:** ^1^ College of Food and Bioengineering, Zhengzhou University of Light Industry, Zhengzhou, China; ^2^ Henan Key Laboratory of Cold Chain Food Quality and Safety Control, Zhengzhou University of Light Industry, Zhengzhou, China

**Keywords:** notch signaling pathway, precision medicine, cancer treatment, immunotherapy, targeted therapy

## Abstract

The Notch signaling pathway and non-coding RNAs (ncRNAs) play significant roles in regulating key cellular processes such as cancer progression, metastasis, and drug resistance. This article systematically reviews the interactions between the Notch pathway and ncRNAs including miRNAs, lncRNAs, and circRNAs, as well as their overall impact on cancer biology. We focus on the latest research progress on how ncRNAs regulate the Notch pathway through transcriptional regulation, post-transcriptional modifications, and epigenetic mechanisms, and discuss how such interactions affect tumor microenvironment shaping, immune escape mechanisms, and treatment sensitivity. Additionally, this article deeply analyzes potential therapeutic strategies targeting the Notch-ncRNA axis, including its prospects for synergistic application with chemotherapy, radiotherapy, and immunotherapy. By integrating multi-cancer experimental data, we propose individualized diagnosis and treatment strategies based on tumor-specific Notch pathway and ncRNA expression patterns.

## 1 Introduction

The Notch signaling pathway is an essential cellular communication mechanism involved in various physiological processes, including embryonic development, immune regulation, and cell fate determination. Initiated by ligand-receptor interactions (e.g., Jagged/Delta ligands binding to Notch1–4 receptors), this pathway triggers γ-secretase-mediated cleavage of the receptor, releasing the Notch intracellular domain (NICD). NICD translocates to the nucleus to activate transcription of target genes such as Hes and Hey, thereby regulating cell proliferation, differentiation, migration, and apoptosis (1).

Emerging evidence highlights the critical role of crosstalk between Notch signaling and non-coding RNAs (ncRNAs), including miRNAs, lncRNAs, and circRNAs, in shaping tumor plasticity and therapeutic resistance. For instance, studies have shown that miR-200 family members can inhibit Notch1 expression by targeting its mRNA, thereby reducing the invasiveness of breast cancer cells (2). Conversely, lncRNA PANDA has been identified to enhance Notch signaling activity in gastric cancer by interacting with the chromatin-modifying complex Polycomb repressive complex 2 (PRC2), promoting tumor growth and metastasis (3).

Moreover, circRNAs have demonstrated their ability to regulate Notch signaling through competing endogenous RNA (ceRNA) mechanisms. For example, circZMIZ1 sponges miR-204, which otherwise inhibits the expression of Notch downstream targets, leading to increased cell proliferation and decreased apoptosis in colorectal cancer cells (4). These bidirectional interactions between Notch signaling and ncRNAs create intricate regulatory networks that significantly influence tumor heterogeneity, immune evasion, and therapeutic resistance.

Deciphering these molecular dialogues holds immense potential for developing novel combinatorial therapies. For instance, RNA-based Notch modulators could be coupled with immune checkpoint inhibitors to enhance the efficacy of cancer treatments while addressing the pathway’s paradoxical roles across different cancer subtypes. By integrating insights from ncRNA-Notch crosstalk into precision oncology strategies, researchers can pave the way for more effective and personalized therapeutic approaches.

In summary, the interplay between Notch signaling and ncRNAs represents a promising avenue for understanding tumor biology and developing innovative treatments. With ongoing advancements in RNA sequencing technologies and functional genomics, further exploration of these interactions will undoubtedly yield valuable insights into cancer pathogenesis and therapeutic resistance mechanisms.

### 1.1 The discovery of the notch signaling pathway

Notch signaling was first discovered in fruit flies (5). In the early 20th century, scientists observed mutants in fruit flies and identified a gene associated with wing defects, which they named “Notch” (6). Later,In 1987, molecular biology studies revealed that the Notch gene encodes a cell surface receptor that participates in cell-to-cell interactions and regulates cell fate determination (7).

**Figure 1:**
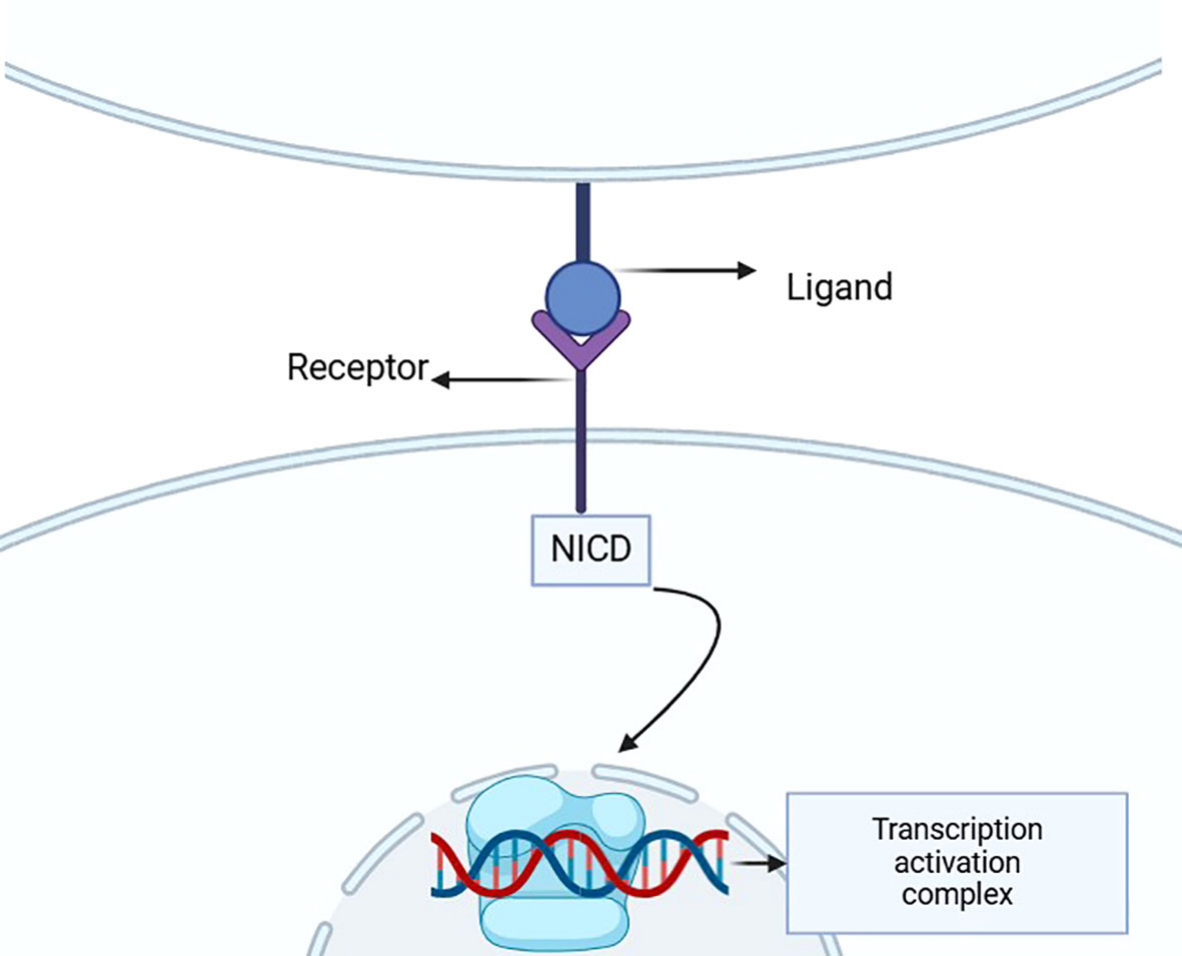
This diagram illustrates the structure of the Notch signaling pathway, highlighting the interaction between the ligand and receptor that triggers the release of NICD. This intracellular domain then translocates to the nucleus, where it activates gene transcription through the transcription activation complex, underscoring its crucial role in developmental processes and disease pathology.

In mammals, the Notch signaling pathway consists of four receptors (Notch1-4) and five ligands (Jagged1, Jagged2, Delta-like 1, Delta-like 3, Delta-like 4) (8–12). When a ligand binds to the receptor, the Notch receptor undergoes a conformational change.Itis then cleaved by proteases such as γ-secretase, releases the Notch intracellular domain (NICD) (1, 10–13). NICD enters the cell nucleus and regulates the transcription of target genes, influencing processes like cell proliferation, differentiation, and migration (14). The Notch signaling plays a crucial role in embryonic development, stem cell maintenance, and immune response (**Figure 1**).

### 1.2 Introduction to non-coding RNAs

ncRNAs are RNA molecules that do not encode proteins but play essential roles in gene expression regulation, cell function, and disease development (15, 16). They are categorized into small ncRNAs (e.g., miRNAs) and long ncRNAs (e.g., lncRNAs, circRNAs), which regulate Notch signaling through epigenetic modifications (e.g., DNA methylation, histone acetylation), transcriptional interference, or post-transcriptional sponging (17), thereby establishing ncRNAs as critical regulators of the Notch pathway.

#### 1.2.1 miRNA

MicroRNAs are small RNA molecules about 20–24 nucleotides in length. They regulate mRNA stability and translation by binding to the 3’ untranslated region (UTR) of target mRNAs (18). miRNAs play a key role in cell proliferation, differentiation, and tumorigenesis. For example, miR-34a directly targets Notch1 mRNA, suppressing its expression and inhibiting breast cancer stem cell self-renewal.

#### 1.2.2 lncRNA

Long non-coding RNAs are RNA molecules longer than 200 nucleotides that regulate gene transcription and translation, and participate in processes such as tumorigenesis and immune regulation (1). LncRNAs like HOTAIR and MALAT1 interact with the Notch signaling pathway to promote tumor cell proliferation, metastasis, and drug resistance (19).

#### 1.2.3 circRNA

Circular RNAs are stable, covalently closed RNA molecules that act as “sponges” for miRNAs, regulating their inhibitory effect on target genes (20). circHIPK3, for instance, sponges miR-124 to enhance Notch1 signaling in hepatocellular carcinoma.

### 1.3 Interaction between notch signaling and non-coding RNAs

The reciprocal regulation between Notch signaling and ncRNAs constitutes a self-reinforcing oncogenic circuit, where epigenetic reprogramming and post-transcriptional control converge to drive disease progression. The complex bidirectional crosstalk between Notch signaling and ncRNAs operates through three-tiered regulatory networks: miRNAs (e.g., miR-34a, miR-7) post-transcriptionally degrade Notch receptors/ligands, lncRNAs (e.g., MALAT1, HOTAIR) scaffold epigenetic modifiers to reshape chromatin accessibility, and circRNAs (e.g., CDR1as) sequester miRNAs or directly bind pathway effectors (21). These multilayered controls collectively amplify Notch-driven pathologies, as exemplified by their stage-specific dysregulation in tumor progression and inflammatory cascades. This regulatory hierarchy establishes ncRNAs as molecular rheostats that fine-tune Notch signaling dynamics, creating therapeutic vulnerabilities discussed in later sections.Below we systematically dissect how each RNA subclass mechanistically hijacks Notch signaling.

#### 1.3.1 miRNAs and notch signaling

As the most studied ncRNA subclass, miRNAs provide rapid feedback control of Notch pathway components.Many miRNAs regulate the activity of Notch signaling by targeting Notch receptors, ligands, or downstream genes. For example, miR-34a acts as a tumor-suppressive miRNA by inhibiting Notch1 expression, thus suppressing tumor cell proliferation and metastasis (22). Notably, miRNA-mediated Notch inhibition often synergizes with chemotherapeutic agents, a concept further explored in Section 6.2.

#### 1.3.2 lncRNAs and notch signaling

LncRNAs orchestrate spatial-temporal control of Notch signaling through chromatin remodeling and protein complex assembly. By interacting with key factors in the Notch signaling pathway (such as NICD)lncRNAs like HOTAIR (HOX transcript antisense RNA) and MALAT1 (metastasis-associated long non-coding RNA transcript 1) promote tumor progression through three-dimensional genomic reorganization (23). Their scaffold function makes lncRNAs ideal targets for epigenetic drugs, as detailed in our therapeutic strategy analysis (Section 6.1-6.3).

#### 1.3.3 circRNAs and notch signaling

CircRNAs establish persistent oncogenic loops by sequestering Notch-regulatory miRNAs. CircRNAs influence signal transduction by sequestering miRNAs or directly regulating key molecules in the Notch signaling pathway, contributing to cancer onset and metastasis.Such as circCDR1as sponges miR-7 to upregulate Notch1, promoting angiogenesis in glioblastoma.The closed-loop structure of circRNAs confers exceptional stability in bodily fluids, a property leveraged in liquid biopsy approaches discussed in Section 6.4.

### 1.4 The role of notch signaling and ncRNAs in cancer and inflammation

Notch signaling and ncRNAs play crucial roles in various diseases, particularly in cancer and inflammatory disorders. Aberrant activation or inhibition of Notch signaling promotes tumor initiation, progression, metastasis, and the development of drug resistance (24). NcRNAs regulate the activity of the Notch pathway, further influencing tumor cell proliferation, metastasis, and immune evasion.

In inflammatory diseases, Notch signaling participates in immune response regulation by modulating immune cell functions. NcRNAs, in turn, regulate immune cell activity, impacting the onset and progression of inflammation. The interplay between Notch signaling and ncRNAs provides new directions and potential targets for treating cancer and inflammatory diseases.

The interactions between Notch signaling and ncRNAs in cancer and inflammation offer fresh perspectives for disease research. A deeper understanding of their interplay is expected to provide new targets and strategies for precision therapy. As the mechanisms of Notch signaling and ncRNAs are further explored, future therapeutic approaches with higher efficacy and specificity may emerge, significantly improving clinical outcomes for cancer and inflammatory disorders.

## 2 The role of notch signaling pathway in cancer

Previous studies have elucidated the interaction mechanisms between the Notch pathway and ncRNAs, highlighting their bidirectional regulatory roles in disease pathogenesis through synergistic or antagonistic effects on cellular fate and pathological progression. Notably, in cancer, aberrant Notch activation/silencing drives malignant transformation by reprogramming tumor proliferation and remodeling the microenvironment, while ncRNAs act as epigenetic regulators to modulate Notch signaling outputs. The following section will focus on the multidimensional roles of Notch in cancer, delineating the molecular networks underlying tumor initiation, stemness maintenance, metastasis, and therapy resistance. Additionally, therapeutic strategies targeting the Notch-ncRNA axis will be evaluated to advance precision oncology.

The role of the Notch signaling pathway in cancer is complex and diverse, as it can both promote tumorigenesis and progression while also inhibit tumor formationThis dual functionality depends on various factors, including cell type, tumor microenvironment, and the specific activation of the signaling pathway (25). Aberrant regulation of the Notch signaling pathway has been shown to play a critical role in cancer occurrence, metastasis, drug resistance, and immune escape across multiple types of malignancies. This section will review the mechanisms by which Notch signaling operates in different tumor contexts, including its role in maintaining tumor stem cells, facilitating tumor metastasis, and contributing to immune evasion.

### 2.1 Notch signaling and tumorigenesis

The abnormal activation or inhibition of the Notch signaling pathway plays a critical role in the onset and progression of various cancers. Notch receptors interact with their ligands, such as Jagged or Delta, to initiate downstream signaling cascades. Subsequently,theintracellular domain of Notch (NICD) translocates into the nucleus where it eitheractivates or represses the transcription of specific target genes (26). Under normal physiological conditions, the Notch signaling pathway mainly regulates cell differentiation, proliferation, and apoptosis. However, in tumor cells, abnormal activation or inhibition of Notch signaling can alter cell fate, thus promoting tumorigenesis and progression (27).

For example, in breast cancer, abnormal activation of Notch1 suppresses the expression of differentiation-related genes (such as GATA3) and drives the upregulation of stem cell-related genes (such as SOX2 and OCT4), This sustains the undifferentiated state of tumor cells, enhances their proliferative capacity, and increasing drug resistance (28, 29). Furthermore, activation not only inhibits the expression of key transcription factors involved in differentiation but also induces the transformation of tumor cells into a cancer stem cell phenotype by upregulating stem cell marker genes like SOX2 and OCT4. This increases the tumor’s self-renewal potential (30, 31). These modifications significantly accelerate tumor initiation and progression, particularly fostering metastasis and recurrence. Additionally, Notch1 activation stimulates tumor cell growth and enhances survival by regulating the expression of cyclins (such as Cyclin D1) and anti-apoptotic proteins (such as Bcl-2) (32).

Although activation of the Notch signaling pathway typically promotes tumor cell proliferation and preserves stem cell characteristics, inhibition of Notch signaling may also induce oncogenic effects in some tumor types. For instance, in pancreatic cancer, inhibition of Notch signaling can suppress tumor cell proliferation and promote differentiation (33). Moreover, in some liver cancers, the inhibition of Notch signaling may result in malignant transformation and tumor progression. This suggests that loss or inhibition of Notch signaling may promote tumor cells to transform into a more aggressive and metastatic phenotype by altering their fate and the tumor microenvironment (34). In these cases, inhibition of Notch signaling may trigger the reprogramming of multiple cellular pathways, causing tumor cells to deviate from normal differentiation pathways, resulting in enhanced proliferation, migration, and drug resistance. Meanwhile, the role of different components of the Notch pathway in the tumor microenvironment also shows significant variability. For example, in glioblastoma, inhibition of Notch signaling helps reduce tumor cell survival, promotes differentiation, and suppresses tumor growth (35). Consequently, the regulation of Notch signaling in different tumor types is influenced not only by the intrinsic characteristics of the cells but also by their interactions with other signaling pathways within the tumor microenvironment.

### 2.2 Notch signaling and cancer stem cells

Cancer stem cells (CSCs) are a special class of cells within tumors that possess self-renewal, differentiation potential, and resistance to therapy. They are the main source of tumor recurrence and metastasis. The Notch signaling pathway plays a key role in maintaining and supporting CSC self-renewal (36). Research has shown that activation of Notch signaling significantly enhances CSC properties, keeping them in an undifferentiated state and providing continuous growth momentum for the tumor (37).

In various types of cancer, including breast cancer, colon cancer, and glioma, abnormal activation or overexpression of Notch signaling is considered a critical factor in maintaining CSC characteristics, promoting tumor growth and conferring therapeutic resistance (38). The Notch pathway, through its classic ligand-receptor interactions (e.g., binding of Notch receptors to Jagged or Delta ligands), activates downstream transcription factors such as Hes1, Hes5, and Hey1, regulating a range of genes related to stem cell fate determination (39). Notch signaling enhances CSC self-renewal and proliferative potential by upregulating stem cell marker genes such as Oct4, Sox2, and Nanog. These genes play essential roles in maintaining the undifferentiated state of stem cells and their proliferation and resistance to therapy (40). Furthermore, Notch signaling may influence cell cycle regulators, anti-apoptotic genes, and DNA repair mechanisms, further enhancing the proliferation capacity of CSCs and their resistance to treatment.

Excessive activation of Notch signaling intersects with other signaling pathways, such as Wnt, Hedgehog, and PI3K/Akt, forming a complex regulatory network that further promotes the survival and functional maintenance of CSCs (41, 42). These signaling pathways act synergistically to enable CSCs to maintain immune escape, proliferation, and drug resistance in the tumor microenvironment, supporting sustained tumor growth.

Therefore, the Notch signaling pathway is a potential therapeutic target for cancer stem cells. Inhibiting Notch signaling activation may help reduce the number of CSCs, thereby slowing tumor recurrence, metastasis, and resistance. Targeting the Notch pathway not only offers a potential strategy for eliminating CSCs but also provides new solutions for addressing drug resistance in cancer treatment.

### 2.3 Notch signaling and tumor metastasis

Tumor metastasis is one of the leading causes of cancer-related death, and the Notch signaling pathway plays a crucial role in this process. Studies have shown that Notch signaling promotes tumor metastasis by regulating tumor cell migration, invasiveness, and the epithelial-mesenchymal transition (EMT) process (43). EMT is a key step for tumor cells to acquire invasiveness and migratory ability. Notch signaling activates a series of transcription factors such as Snail, Slug, and Twist, triggering EMT, which drives tumor cells to transform into a more invasive and metastatic phenotype (44).

In several types of cancer, including lung, gastric, and pancreatic cancer, activation of Notch signaling has been shown to significantly promote the EMT process, enhancing tumor cell migration and invasiveness (45, 46). Notch signaling, by receptor-ligand binding, activates downstream transcription factors that regulate the expression of genes associated with EMT, such as Snail, Slug, and Twist. The upregulation of these transcription factors leads to the loss of cell polarity, disruption of intercellular junctions, and cytoskeletal remodeling, thereby enabling tumor cells to acquire invasive and migratory properties (47). This process allows tumor cells to break through the boundary of the primary tumor, spread to surrounding tissues, and eventually form metastatic lesions.

Additionally, Notch signaling regulates components of the extracellular matrix (ECM) in the tumor microenvironment, further enhancing tumor cell invasive abilities. Activation of Notch signaling may alter the structure and composition of the ECM, regulating the interaction between tumor cells and the matrix, which in turn enhances tumor cell adhesion, migration, and invasion abilities (48, 49). The close interaction between tumor cells and the matrix is crucial for metastasis; Notch signaling promotes this interaction process, aiding tumor cells in breaking away from the primary tumor and entering the bloodstream or lymphatic system, ultimately leading to distant metastasis (50).

Therefore, inhibiting Notch signaling may weaken tumor cells’ migration and invasion abilities by blocking the EMT process, thus effectively reducing the formation of cancer metastasis. Targeting Notch signaling is expected to provide new therapeutic approaches for the prevention and treatment of cancer metastasis.

### 2.4 Notch signaling and tumor immune escape

The ability of tumor cells to escape immune system surveillance is one of the key factors contributing to tumor progression, metastasis, and the development of drug resistance (51). Increasing evidence shows that Notch signaling plays an important role in tumor immune escape (52). Notch signaling regulates immune cell function, alters immune responses in the tumor microenvironment, and facilitates immune escape of tumor cells (53, 54).

An important mechanism by which Notch signaling promotes tumor immune escape is by inducing the polarization of tumor-associated macrophages (TAMs) into the M2 phenotype (55). M2 macrophages have strong immunosuppressive effects and can inhibit T cell activation, thereby providing an immune escape advantage for tumor cells. Studies have shown that activation of Notch signaling drives TAM polarization into the M2 phenotype, creating an immunosuppressive microenvironment that enhances tumor cell growth and metastasis (56). This process weakens anti-tumor T cell responses and promotes immune escape of tumor cells.

In addition to regulating immune cells, Notch signaling also promotes immune escape by modulating the expression of immune checkpoint moleculessuch as PD-L1, on the surface of tumor cells (57). Research has shown that activation of Notch signaling significantly upregulates PD-L1 expression in tumors like breast cancer, lung cancer, and melanoma (58). PD-L1 binds to the PD-1 receptor on T cells, inhibiting T-cell immune responses and reducing tumor immune surveillance (59). Through this mechanism, tumor cells can effectively escape recognition and elimination by the host immune system.

Therefore, targeting the Notch signaling pathway may offer novel strategies for enhancing immune system surveillance of tumors and improving immunotherapy outcomes. By inhibiting abnormal Notch signaling activation, it is possible to reduce immunosuppressive elements in the tumor microenvironment, enhance immune cell recognition and cytotoxicity toward tumor cells, and ultimately improve therapeutic efficacy.

### 2.5 Notch signaling and tumor drug resistance

Tumor cell resistance to therapy is a major challenge in clinical cancer treatment. The abnormal activation of the Notch signaling pathway has been found to be closely related to tumor drug resistance (60). Studies show that Notch signaling enhances tumor cell resistance to chemotherapy and targeted therapy by regulating mechanisms such as cell proliferation, apoptosis, and DNA repair (61). For example, activation of Notch1 can inhibit apoptosis by upregulating anti-apoptotic proteins (such as Bcl-2 and Survivin), thereby increasing tumor cell survival (62). Furthermore, Notch signaling enhance tumor resistance by promoting the proliferation and self-renewal of cancer stem cells.

In cancers such as breast cancer, pancreatic cancer, and non-small-cell lung cancer, abnormal activation of Notch signaling is closely associated with chemotherapy resistance. Therefore, inhibiting Notch signaling or targeting downstream molecules associated with the Notch pathway could become a new strategy to overcome tumor drug resistance.

In summary, the role of Notch signaling in cancer is complex and multifaceted. It directly influences tumor initiation and progression by regulating various aspects of tumor cell behavior, including proliferation, differentiation, migration, immune escape, and drug resistance. As our understanding of the mechanisms underlying Notch signaling deepens, targeting the Notch pathway is expected to offer new therapeutic approaches for cancer treatment. Future research should further explore the interactions between Notch signaling and other cellular pathways to develop treatment strategies with greater clinical application potential.

## 3 The role of the notch signaling pathway in inflammation

The Notch pathway’s dual regulatory capacity in cancer - governing stemness maintenance and immune evasion - mirrors its pivotal role in inflammatory pathogenesis. Crucially, Notch-mediated control of macrophage polarization and T-cell differentiation, central to creating immunosuppressive tumor microenvironments, is evolutionarily repurposed to drive chronic inflammation through analogous immune cell reprogramming. This functional conservation establishes Notch as a molecular linchpin connecting oncogenic and inflammatory processes through shared mechanisms of cellular fate determination.

**Figure 2:**
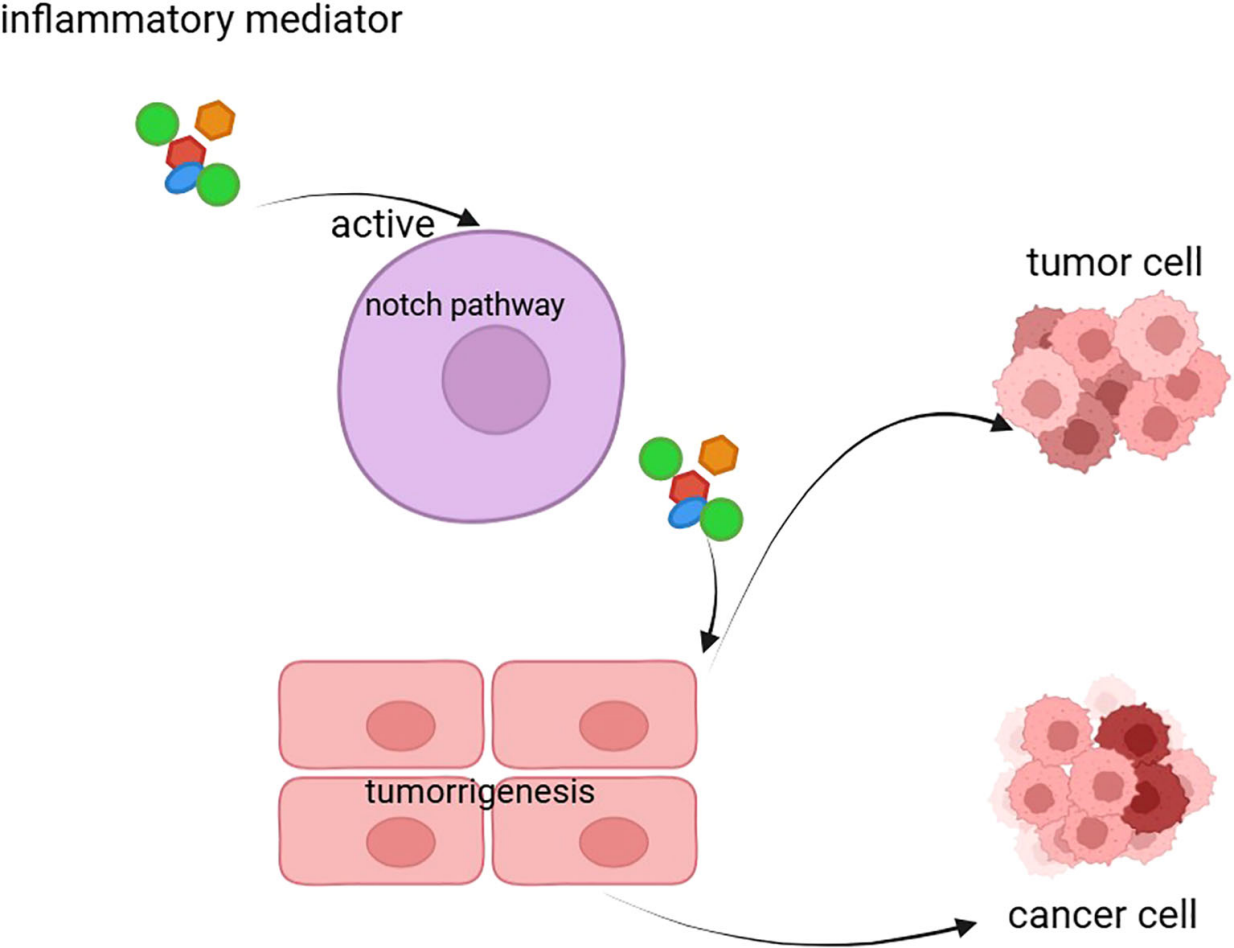
The figure illustrates the inflammation-driven Notch signaling pathway that leads to tumorigenesis and cancer. Inflammatory mediators activate the Notch pathway, which promotes tumor cell proliferation and transformation into cancer cells. This process highlights the role of inflammation in driving the progression from normal cells to malignant states through the Notch signaling mechanism.

The Notch signaling pathway plays a critical role not only in cell development and tumor progression but also in immune regulation and inflammatory responses. As a highly conserved intercellular communication pathway, Notch signaling regulates the differentiation, function, and activity of immune cells through the interactions between its receptors and ligands, thereby influencing the onset and progression of inflammatory responses (**Figure 2**) (63).

Recent studies have demonstrated that the Notch signaling pathway plays pivotal roles in both chronic inflammatory diseases (such as rheumatoid arthritis and inflammatory bowel disease) and acute inflammatory responses (64). This article will explore in detail the role of Notch signaling in inflammation and its potential therapeutic implications.

### 3.1 The role of the notch signaling pathway in immune cells

The Notch signaling pathway regulates immune responses in various immune cells, with its function depending on the cell type and microenvironment (27). In immune cells such as T cells, B cells, macrophages, and dendritic cells, the activation or inhibition of the Notch pathway directly influences their function and differentiation processes.

#### 3.1.1 Notch signaling in T cells

Notch signaling plays a crucial role in T cell development and function. During T cell development, the Notch pathway regulates the fate of T cell precursors in the thymus, promoting their differentiation into different subsets, such as CD4+ and CD8+ T cells (65). Studies have also shown that Notch signaling is pivotal in T cell activation and differentiation, particularly in regulating the balance between regulatory T cells (Tregs) and effector subsets (Th1/Th17) (66).

In inflammatory processes, Notch signaling contributes to inflammation by promoting Th17 cell differentiation. Th17 cells secrete cytokines such as IL-17, which exacerbate tissue damage and inflammatory responses (67). In chronic inflammatory diseases like rheumatoid arthritis, aberrant activation of the Notch pathway can enhance Th17 cell function, leading to dysregulated immune activation and tissue injury (68). Thus, modulating the activity of the Notch pathway activity can regulate Th17 cell differentiation and alleviate chronic inflammation.

#### 3.1.2 Notch signaling in macrophages

Macrophages are key effector cells in inflammatory responses, capable of regulating local immune reactions through the secretion of cytokines and chemokines. The Notch signaling pathway plays an important role in macrophage polarization, influencing their transition into different subtypes, such as M1 and M2 macrophages (69).

In acute inflammatory responses, M1 macrophages promote inflammation by secreting pro-inflammatory cytokines such as TNF-α and IL-1β. Conversely, in chronic inflammation, M2 macrophages secrete anti-inflammatory cytokines such as IL-10 and TGF-β, exerting immunosuppressive effects (70, 71).

Notch signaling regulates macrophage polarization. For example, Notch1 activation promotes M1 macrophage differentiation, enhancing inflammatory responses. In contrast, Notch2 activation tends to promote M2 macrophage generation, thereby reducing inflammation (72). These findings suggest that targeting the Notch signaling pathway could serve as a crucial target for modulating inflammatory responses.

#### 3.1.3 Notch signaling in dendritic cells

Dendritic cells (DCs) are essential antigen-presenting cells in the immune system that prime T cell-mediated immune responses (73). The Notch signaling pathway plays a critical role in DC development and functional regulation. Notch signaling not only regulates DC maturation but also determines the polarization of T cell responses by influencing their cytokine profiles. For instance, Notch signaling can drive DC polarization towards a Th17 response, thereby exacerbating inflammation (74, 75).

### 3.2 The role of notch signaling in chronic inflammation

Chronic inflammatory diseases, such as rheumatoid arthritis (RA), inflammatory bowel disease (IBD), and systemic lupus erythematosus (SLE), are closely linked to prolonged immune system activation and tissue damage (76). In these conditions, dysregulated activation or suppression of the Notch signaling pathway may contribute to immune cell imbalance and persistent inflammation.

#### 3.2.1 Rheumatoid arthritis

In RA, abnormal activation of the Notch signaling pathway is strongly associated with sustained inflammation and joint damage. Studies have shown that excessive activation of Notch1 promotes the activation of T cells and macrophages in joint tissues, leading to exacerbated inflammatory responses. Additionally, Notch signaling may participate in joint destruction by regulating stem cell behavior in the bone marrow (77). Targeting the Notch signaling pathway could therefore provide new therapeutic approaches for RA.

#### 3.2.2 Inflammatory bowel disease

IBD encompasses chronic inflammatory disorders involving abnormal activation of the intestinal immune system (78). Research indicates that Notch signaling plays a significant role in the onset and progression of IBD. Activation of Notch1 can enhance Th17 cell function and inflammatory responses, leading to persistent damage to intestinal tissues. Conversely, Notch2 exhibits anti-inflammatory effects by suppressing Th1/Th17 cell differentiation (79). The dual roles of Notch signaling in IBD make it a promising therapeutic target.

### 3.3 Notch signaling and acute inflammatory response

The acute inflammatory response is a rapid immune reaction to infection or injury aimed at eliminating pathogens and repairing damaged tissues. Notch signaling plays a complex role in acute inflammation, capable of both promoting the inflammatory response and preventing excessive immune activation by regulating immune cell functions (80).

In acute inflammation, Notch signaling modulates immune cell activity, such as that of T cells and macrophages, to rapidly respond to pathological signals. For example, Notch1 activation during acute lung injury enhances macrophage chemotaxis and phagocytosis, aiding in pathogen clearance and tissue repair (81). Simultaneously, Notch signaling regulates immune cell apoptosis and regeneration to prevent tissue damage caused by excessive inflammation.

### 3.4 Targeted therapies for the notch signaling pathway

Given its critical role in inflammatory responses, targeting the Notch signaling pathway has emerged as a promising therapeutic intervention for treating inflammatory diseases. Studies have explored the potential of alleviating symptoms of chronic inflammatory diseases by inhibiting Notch activity. For instance, Notch inhibitors have demonstrated anti-inflammatory effects in animal models of RA and IBD (82).

However, the pleiotropic regulatory effects of Notch signaling suggest that inhibition might lead to immunosuppression in some cases, increasing the risk of opportunistic infections (83). Thus, precisely modulating Notch signaling, particularly in specific immune cell subsets, remains a crucial challenge in the treatment of inflammatory diseases.

The role of the Notch signaling pathway in inflammation is functionally multifaceted, influencing immune cell differentiation and function as well as the development and progression of chronic inflammatory diseases. Through deeper investigations into the specific mechanisms of Notch signaling in various immune cells and its involvement in inflammatory responses, targeted therapies for inflammatory diseases may become more effective. As understanding of the Notch pathway advances, precise modulation of its role in immune responses will provide novel strategies for clinical treatment of inflammatory conditions.

## 4 The role of non-coding RNA in cancer

NcRNAs play a crucial role in regulating gene expression, cell proliferation, and apoptosis through their interactions with DNA, RNA, and proteins. These processes are closely related to tumor initiation, progression, metastasis, and drug resistance, making ncRNAs significant in cancer biology and therapy (**Table 1**) (84).

**Table 1:** Roles of non-coding RNA in cancer.

Type of ncRNA	Name	Cancer Type	Description of Role	References
miRNA	miR-34a	Prostate cancer	Promotes apoptosis, targets anti-apoptotic genes like Bcl-2	(85)
miRNA	miR-21	Breast,lung, colorectal cancer	Promotes proliferation, inhibits apoptosis, linked to poor prognosis	(86)
miRNA	miR-10b	Brain cancer	Facilitates tumor cell migration and invasion	(87)
miRNA	miR-122	Liver cancer	Regulates liver metabolism and cell proliferation	(88)
miRNA	miR-145	Colorectal cancer	Inhibits tumor cell proliferation and invasion	(89)
miRNA	miR-146a	Breast cancer	Regulates cell proliferation and inflammatory response	(90)
lncRNA	HOTAIR	Breast,liver cancer	Promotes tumor metastasis, regulates chromatin remodeling	(91)
lncRNA	MALAT1	Lung cancer	Associated with tumor invasion and metastasis	(92)
circRNA	circHIPK3	Liver cancer	Inhibits proliferation, induces apoptosis	(93)

### 4.1 miRNAs in cancer

MiRNAs are small ncRNAs that regulate gene expression by binding to the 3’ untranslated regions (UTRs) of target mRNAs, leading to translational repression or degradation (94). Their roles in cancer are diverse, functioning either as tumor suppressors or oncogenes (95).

#### 4.1.1 Tumor-suppressive miRNAs

Some miRNAs are downregulated or lost in cancer cells, playing tumor-suppressive roles. For example, miR-34a directly targets Notch1, inhibiting tumor cell proliferation, migration, and invasion (96). Reduced miR-34a expression is linked to various cancers, including breast, lung, and liver cancers, while restoring miR-34a can significantly inhibit tumor growth and metastasis (97).

miR-143 and miR-145 are also considered tumor-suppressive miRNAs, regulating tumor-related genes like RAS, ERK, and AP-1 to inhibit proliferation and metastasis (98). Their low expression is often associated with increased malignancy, highlighting their critical roles in tumor suppression.

#### 4.1.2 Oncogenic miRNAs

Conversely, some miRNAs act as oncogenes, being upregulated in cancers. For instance, miR-21 is widely recognized as an oncogene, and its high expression is closely associated with tumor initiation and progression (99).miR-21 promotes proliferation, survival, and invasion by inhibiting target genes like PTEN and PDCD4 (100).

Other oncogenic miRNAs include miR-155 and miR-221/222. miR-155 enhances proliferation and migration by targeting suppressor genes like SOCS1 (101), while miR-221/222 promotes tumor progression by regulating cell cycle inhibitors like p27kip1 (102).

### 4.2 lncRNAs in cancer

lncRNAs participate in chromatin remodeling, transcriptional control, and signal transduction (103). Their roles in cancer vary, promoting tumor progression in some cases while inhibiting it in others.

#### 4.2.1 Oncogenic lncRNAs

Some lncRNAs are overexpressed in cancer cells, promoting proliferation, metastasis, and drug resistance by regulating tumor suppressor genes, oncogenes, or epigenetic factors. For example, HOTAIR is overexpressed in several cancers, recruiting Polycomb Repressive Complex 2 (PRC2) and other epigenetic regulators to suppress tumor suppressor genes, thereby facilitating proliferation and invasion (104).

MALAT1, another oncogenic lncRNA, is highly expressed in various cancers. It regulates transcription factors and RNA-binding proteins, affecting cell cycle progression, transcription, and migration, thus promoting metastasis (105). High MALAT1 expression correlates with poor prognosis, making it a potential therapeutic target.

#### 4.2.2 Tumor-suppressive lncRNAs

However, the down-regulation of certain lncrnas is associated with the development of cancer. A typical example is TUSC7 (Tumor Suppressor Candidate 7), which has low expression in multiple tumor types, and restoring the expression of TUSC7 can significantly inhibit the proliferation and metastasis of tumor cells (106). Mechanistically, TUSC7 coordinates tumor suppression through direct interaction with p53 protein and sequestration of oncogenic miRNAs (107).

Another tumor-suppressive lncRNA, p53-Activated Noncoding RNA (PANDA), modulates cancer cell fate by stabilizing wild-type p53 protein, thereby inducing cell cycle arrest and apoptotic signaling cascades (108). The low expression of PANDA is associated with the development of various cancers, and restoring its expression is expected to be one of the therapeutic strategies.

### 4.3 circRNAs in cancer

circRNAs characterized by their unique closed-loop structure, are stable ncRNAs that act as miRNA sponges, regulating gene expression. Abnormal circRNA expression is closely associated with tumor initiation, progression, and metastasis (109).

For instance, circRNA CDR1as binds to miR-7, preventing it from targeting its gene. This interaction promotes tumor proliferation and migration, with high CDR1as levels being linked to poor prognosis (110). Similarly, circHIPK3 regulates cancer cell proliferation and invasion through interactions with miR-124-3p and other molecules, making it a promising biomarker for cancer diagnosis and therapy (111).

### 4.4 NcRNAs in cancer therapy

The critical roles of ncRNAs in cancer have made them attractive therapeutic targets. Strategies targeting miRNAs, lncRNAs, and circRNAs are being developed. Restoring tumor-suppressive miRNAs or inhibiting oncogenic miRNAs has shown promise in suppressing proliferation and metastasis (112). Similarly, therapies targeting specific lncRNAs and circRNAs are gaining traction in cancer research.

Despite their potential, ncRNA-based clinical applications face challenges. The complexity of ncRNAs and their heterogeneity across tumor types pose difficulties in precise targeting while minimizing side effects. Advancing our understanding of ncRNA mechanisms could lead to more efficient and specific therapies in the future.

In conclusion, ncRNAs play pivotal roles in oncogenesis. miRNAs, lncRNAs, and circRNAs regulate tumor initiation, progression, and metastatic dissemination through diverse mechanisms. Understanding these roles offers new avenues for early diagnosis, prognosis, and individualized therapy. With continued research, ncRNA-based therapies could become vital tools in cancer treatment.

## 5 Interaction between notch signaling and ncRNAs

NcRNAs serve as molecular hubs in cancer by orchestrating complex regulatory networks, yet their functional impact hinges on dynamic interactions with key signaling pathways. Among these, the Notch signaling pathway—a master regulator of cell fate and tumor progression—has been identified as a critical partner of n cRNAs. Research demonstrates that miRNAs, lncRNAs, and circRNAs directly modulate Notch receptor activity or indirectly influence downstream effectors through epigenetic reprogramming and feedback loops, establishing bidirectional crosstalk. This interplay not only fuels tumor heterogeneity, microenvironment remodeling, and therapy resistance but also unveils opportunities to overcome the limitations of conventional single-target therapies. The following section delves into the Notch-ncRNA axis, dissecting its mechanistic roles in cancer progression and its potential for precision oncology.

The Notch signaling pathway governs fundamental cellular processes, including proliferation, differentiation, and apoptosis. Mounting evidence highlights its intricate crosstalk with ncRNAs, which fine-tune Notch activity at multiple levels. By regulating pathway components or interacting with epigenetic modifiers, ncRNAs shape Notch-driven phenotypes in cancer and inflammatory diseases. These discoveries not only deepen our understanding of disease mechanisms but also pave the way for innovative therapeutic strategies targeting the Notch-ncRNA axis.

### 5.1 Overview of the notch signaling pathway

Abnormal activation of the Notch signaling pathway is closely associated with various diseases, including cancer, cardiovascular diseases, and immune disorders. In cancer, in particular, Notch activation often leads to tumor proliferation, metastasis, and drug resistance and is also linked to the maintenance of cancer stem cells.

### 5.2 Interaction between miRNAs and the notch signaling pathway

miRNAs are essential molecules for regulating gene expression. They typically bind to the 3’ untranslated regions (3’ UTRs) of target mRNAs, thereby suppressing their stability and translation. miRNAs play critical roles in regulating the Notch signaling pathway. They can inhibit the expression of Notch receptors to reduce pathway activation or activate the Notch pathway to promote cell proliferation and migration (113).

#### 5.2.1 miRNAs inhibiting notch signaling

For instance, miR-34a targets Notch1, suppressing its expression and negatively regulating the Notch signaling pathway. Low expression of miR-34a is often associated with tumor malignancy, metastasis, and poor prognosis. Thus, miR-34a exhibits potential anti-tumor effects, and restoring miR-34a expression can significantly inhibit cancer cell proliferation and migration (114).

Similarly, miR-7, miR-196a, and miR-129 have also been found to inhibit Notch signaling by directly suppressing the expression of Notch receptors or their transcriptional activators (115). For example, miR-7 targets Notch1 mRNA, suppressing its expression and thereby reducing Notch signaling activity in non-small cell lung cancer (116). Furthermore, miR-196a, which is upregulated in gastric cancer, downregulates Notch2 expression, inhibiting the proliferation and invasive potential of gastric cancer cells (117).

#### 5.2.2 miRNAs activating notch signaling

In contrast to the inhibitory effects of the aforementioned miRNAs, certain miRNAs can promote the activation of Notch signaling, thereby facilitating cancer cell proliferation and metastasis. For instance, miR-221/222 is upregulated in various cancers and promotes tumor cell proliferation and migration by targeting cell cycle regulators such as p27kip1 (118).

Moreover, miR-9 has been shown to regulate Notch signaling, supporting the self-renewal of embryonic stem cells and contributing to tumorigenesis. miR-9 enhances Notch signaling in breast cancer by targeting and upregulating Notch3, which drives breast cancer progression (119).

### 5.3 Interaction between lncRNAs and notch signaling

LncRNAs play vital roles in gene transcription, epigenetic modification, and cellular biological processes. Many lncRNAs interact with key factors in the Notch signaling pathway to regulate its activity, thereby influencing the onset and progression of cancer.

#### 5.3.1 lncRNAs inhibiting notch signaling

Some lncRNAs suppress the Notch signaling pathway by directly or indirectly regulating the expression of Notch receptors (120). For example, lncRNA HOTAIR binds to the Notch1 receptor and inhibits its signal transduction, thereby promoting malignancy in various cancers. High expression of HOTAIR is closely associated with tumor invasiveness, metastasis, and poor prognosis (121).

Similarly, lncRNA ANRIL (Antisense Noncoding RNA in the INK4 Locus) interacts with critical factors in the Notch signaling pathway to suppress its activity, resulting in tumor cell proliferation and metastasis (122, 123).

#### 5.3.2 lncRNAs activating notch signaling

In contrast to tumor-suppressive lncRNAs, certain long non-coding RNAs function as oncogenic drivers by potentiating Notch signaling transduction (124). A prime example is the metastasis-associated lung adenocarcinoma transcript 1 (MALAT1), which exhibits pathological overexpression across malignancies and directly binds to Notch1 mRNA, thereby stabilizing its transcript and enhancing Notch signaling output. This molecular interplay promotes clonal expansion, metastatic dissemination, and chemoresistance phenotypes in neoplastic cells (125). Mechanistically, MALAT1 orchestrates a multi-layered regulatory network through:1. Transcriptional upregulation of Notch1 via chromatin remodeling complexes. 2. Sponging tumor-suppressive miRNAs (e.g., miR-129-5p). 3.Recruiting epigenetic modifiers (DNMT3A/HDAC1) to reprogram Notch-responsive genes.

Similarly, terminal differentiation-induced non-coding RNA (TINCR) demonstrates oncogenic competence in melanoma, where its ectopic overexpression activates Notch signaling via ligand-independent receptor clustering, consequently augmenting cancer cell motility and matrix-invasion capacity (126).

### 5.4 Interaction between circRNAs and notch signaling

Circular RNAs (circRNAs) are a class of ncRNAs with a covalently closed loop structure. Due to their stability and regulatory functions, circRNAs have become a focus of cancer research in recent years. CircRNAs regulate the suppression of target genes by acting as miRNA sponges. Additionally, circRNAs can directly interact with key molecules in the Notch signaling pathway, further modulating tumor initiation and progression (127).

#### 5.4.1 circRNAs regulating notch signaling

For example, circRNA CDR1as (Cerebellar Degeneration-Related Protein 1-antisense RNA) acts as a sponge for miR-7, relieving miR-7’s inhibitory effect on Notch1, thereby activating Notch signaling and promoting tumor cell proliferation and migration. The high expression of CDR1as in gliomas exacerbates tumor malignancy through this mechanism (128). Similarly, circHIPK3 enhances Notch signaling activity by regulating molecules like miR-124, driving tumor growth and metastasis (129). The high expression of circRNA circPVT1 in gastric cancer cells activates Notch signaling by binding to miR-125b, promoting cancer cell proliferation (130).

### 5.5 Bidirectional regulation between notch signaling and ncRNAs

The interaction between Notch signaling and ncRNAs is highly complex. Not only do they mutually regulate each other during signal transduction, but they can also form feedback mechanisms to ensure precise control of cells under various physiological and pathological conditions. Activation of Notch signaling can upregulate the expression of miRNAs and lncRNAs, which in turn influence the regulation of Notch signaling by these ncRNAs. On the other hand, ncRNAs directly bind to Notch receptors or downstream effector molecules, modulating the strength and duration of Notch signaling.

For instance, activation of Notch1 can upregulate the expression of miR-145, which in turn forms a negative feedback loop by inhibiting Notch1’s target genes. In tumor cells, this feedback mechanism helps maintain a balance between cell proliferation and differentiation (131). This bidirectional regulatory mechanism ensures that cells respond accurately to external signals and adapt to changes in their internal environment. In the tumor microenvironment, the interaction between Notch signaling and ncRNAs is especially critical, as they jointly regulate tumor cell proliferation, migration, immune evasion, and drug resistance (**Table 2**).

**Table 2:** The interregulation of notch signaling and ncRNAs.

Type of ncRNA	Name	Cancer Type	Description of Role	Regulation of Notch Signaling	References
miRNA	miR-34a	Various cancers	By targeting Notch1 mRNA, it inhibits the Notch signaling pathway, affecting cell proliferation and apoptosis	Inhibits Notch1 expression	(96)
lncRNA	HOTAIR	Various cancers	By interacting with Notch1, it enhances the Notch signaling pathway, promoting tumor metastasis and invasion	Enhances Notch signaling	(132)
lncRNA	MALAT1	Lung cancer	By regulating the Notch signaling pathway, it affects the proliferation and metastasis of lung cancer cells	Regulates Notch signaling	(133)
circRNA	circHIPK3	Liver cancer	By sponge absorption of miRNAs, it indirectly upregulates genes related to the Notch signaling pathway, influencing liver cancer cell proliferation	Indirectly upregulates Notch signaling	(134)
lncRNA	LINC00982	Gastric cancer	By interacting with the Notch1 receptor, it promotes the proliferation and invasion of gastric cancer cells	Promotes Notch signaling	(135)
miRNA	miR-200 family	Ovarian cancer	By targeting Notch pathway-related molecules like DLL4, it regulates the invasion and metastasis of ovarian cancer cells	Regulates Notch pathway	(136)

## 6 Therapeutic strategies and prospects

The reciprocal regulatory axis between ncRNAsand the Notch signaling pathway underscore their central role in cancer biology, yet the translational potential of this complex network remains underexplored. Current studies demonstrate that dynamic Notch-ncRNA interactions not only drive malignant phenotypes but also modulate therapeutic responses through epigenetic landscape remodeling and immune microenvironment remodeling. However, translating these mechanisms into clinical applications faces dual challenges: the “double-edged sword” nature of Notch signaling necessitates balancing antitumor efficacy with preservation of normal tissue functions in targeted therapies, while the spatiotemporal specificity of ncRNA expression demands innovations in spatiotemporally controlled delivery platforms and tissue-selective intervention strategies. The sixth section will systematically elaborate on novel therapeutic approaches based on these molecular mechanisms and explore the potential of multimodal combination therapies to overcome therapeutic resistance and enhance efficacy, thereby expanding the horizons of precision oncology.

With the progressive elucidation of Notch-ncRNA interactions, researchers have prioritized therapeutic strategies targeting this axis. Notch signaling and ncRNAs not only show immense promise in cancer therapy but also offer potential therapeutic targets for addressing chronic inflammation, immune evasion, and drug resistance (60). The following sections will delve into these strategies, focusing on advancements in targeted therapy, immunotherapy, and RNA-based interventions.

### 6.1 Targeting notch signaling pathway in therapy

The abnormal activation or inhibition of Notch signaling plays a critical role in the initiation and progression of various cancers. Therefore, Notch signaling has become an important target in cancer therapy. Current therapeutic strategies mainly include small molecule inhibitors, monoclonal antibodies, and γ-secretase inhibitors.

#### 6.1.1 Small molecule inhibitors

A key regulator of Notch signaling is γ-secretase, which cleaves the Notch receptor, releasing the Notch intracellular domain (NICD) and activating downstream signaling pathways (137). Therefore, γ-secretase inhibitors (such as DAPT, LY3039478, etc.) are considered potential inhibitors of Notch signaling (138). These inhibitors have shown certain therapeutic effects in clinical trials, particularly in hematologic malignancies (e.g., acute myeloid leukemia).

#### 6.1.2 Monoclonal antibodies

Monoclonal antibodies targeting Notch ligands such as Jagged1, Jagged2, and Delta-like can specifically block the binding of Notch receptors to their ligands, thereby inhibiting the activation of Notch signaling (139) Studies have found that monoclonal antibodies targeting Jagged1 and Jagged2 also exhibit tumor growth inhibitory effects in certain cancer types.

#### 6.1.3 Notch receptor targeted

Therapeutic Strategies: Preclinical studies demonstrate that selective anti-Notch1 monoclonal antibodies (e.g., Omp-18) can significantly attenuate tumor cell proliferative capacity and metastatic dissemination through ligand-binding domain blockade. Concurrently, isoform-specific oncogenic roles of Notch3 in hormone receptor-positive breast cancer and Notch4 in non-small cell lung adenocarcinoma have been mechanistically validated, revealing therapeutic vulnerabilities for precision intervention.Notwithstanding these advances, clinical translation faces two fundamental challenges:1.Pathway complexity: The pleiotropic nature of Notch signaling necessitates tumor-specific isoform inhibition to avoid homeostatic disruption in normal tissues.2.Therapeutic window optimization: On-target toxicity arising from conserved receptor-ligand interactions in stem cell niches limits dose escalation

Future investigations must prioritize tumor microenvironment (TME)-responsive delivery systems capable of spatial-temporal modulation and single-cell resolution targeting. Emerging approaches combining γ-secretase modulators with Notch extracellular domain (NECD) decoys may enable context-dependent signaling attenuation while preserving physiological Notch functions.

### 6.2 Non-coding RNA-based therapeutic strategies

NcRNAs, particularly miRNAs, lncRNAs, and circRNAs, play essential roles in the fine-tuning of gene expression, influencing various biological behaviors of cancer cells, such as proliferation, migration, and immune evasion. Therapeutic strategies based on ncRNAs mainly include miRNA replacement therapy, RNA intervention technologies, and the regulation of lncRNAs and circRNAs to improve the clinical treatment of cancer.

#### 6.2.1 miRNA replacement therapy

Many miRNAs are underexpressed or dysregulated in cancer, leading to abnormal cancer cell proliferation and metastasis. By introducing specific miRNAs (e.g., miR-34a, miR-143) into cancer cells, the normal function of miRNAs can be restored, inhibiting tumor growth and enhancing sensitivity to chemotherapy (140).

#### 6.2.2 RNA intervention technologies

In addition to miRNA replacement therapy, RNA intervention technologies (such as small molecule RNAs, siRNAs, etc.) have become a recent research focus in cancer therapy. By designing specific small molecule RNAs, key gene expressions in cancer cells can be precisely regulated. These technologies can inhibit key molecules in the Notch signaling pathway, such as Jagged and Notch receptors, interfering with cancer cell proliferation and migration. Furthermore, RNA intervention technologies can also target ncRNAs, such as lncRNAs and circRNAs, to regulate intracellular signaling pathways, further controlling cancer development.

#### 6.2.3 lncRNA and circRNA regulatory therapy

Abnormal expression of lncRNAs and circRNAs is widespread in cancer. Therefore, intervening in the expression of these ncRNAs may provide new strategies for cancer therapy. Researchers are developing inhibitors or agonists targeting specific lncRNAs (e.g., HOTAIR, MALAT1) and circRNAs (e.g., circRNA CDR1as) to restore or suppress their functions, inhibiting cancer cell proliferation and metastasis (141).

### 6.3 Immunotherapy-based strategies

Immunotherapy, as an emerging field in cancer treatment, has made significant progress in recent years. Notch signaling not only regulates cancer cell proliferation and migration but also plays an important role in immune evasion. As a result, immunotherapy targeting the Notch signaling pathway has become a research focus.

#### 6.3.1 Immune checkpoint inhibition

The role of Notch signaling in immune responses suggests that regulating the Notch pathway can improve immune evasion. Studies have found that the Notch pathway plays a significant role in the function of T cells and B cells, and targeting Notch with therapeutic agents may enhance the immune system’s ability to recognize and eliminate tumors (142). Combining immune checkpoint inhibitors with Notch signaling pathway inhibitors may improve the effectiveness of cancer immunotherapy.

### 6.4 Clinical translation of notch pathway inhibitors: recent advances and challenges

The therapeutic development of Notch pathway inhibitors has achieved substantial clinical progress, with over 30 interventional trials initiated since 2022 demonstrating improved safety and efficacy profiles. Second-generation γ-secretase inhibitors (GSIs) exemplify these advancements, as evidenced by Nirogacestat (NCT05348356) achieving breakthrough designation with a 76% objective response rate in desmoid tumors through optimized pulsatile dosing that mitigates gastrointestinal toxicity (143). This progress extends to receptor-specific biologics, where Brontictuzumab elicited partial responses in 15% of NOTCH1-mutated solid tumors, and Tarextumab combined with chemotherapy extended progression-free survival by 5.1 months in pancreatic cancer (NCT01647828) (144, 145).

Innovative molecular approaches are addressing historical limitations in Notch modulation. The transcriptional inhibitor CB-103 disrupts NICD/RBPJκ complex formation, achieving 40% disease control in adenoid cystic carcinoma while avoiding dose-limiting intestinal toxicity (146). Combination therapies demonstrate synergistic potential, particularly in AL101 plus Pembrolizumab (NCT05608762) for HNSCC, where PD-L1 suppression and T-cell activation drove a 38% overall response rate (147). These therapeutic advances are complemented by liquid biopsy applications, with ctDNA analysis of NOTCH1/FBXW7 mutations achieving 92% concordance with tissue biopsies in T-ALL for real-time treatment monitoring (148).

Persisting challenges in pathway pleiotropy and context-dependency are driving biomarker development. Current strategies integrate 68Ga-Notch4i PET imaging (SUVmax ≥2.5 predictive of response) with single-cell RNA sequencing to delineate tumor-specific Notch activity patterns (149). Pharmaceutical innovation continues with microenvironment-responsive agents, including pH-sensitive GSI formulations and Notch3-targeted CAR T-cell platforms (150). The field is poised for transformative growth, with 18 novel modulators entering clinical trials between 2023-2024, underscoring the evolution toward precision-guided interventions through integrated systems biology and multi-omics profiling (**Table 3**).

**Table 3:** Overview of drugs and their clinical characteristics.

Drug Name	Target/Mechanism	Clinical Stage	Key Results (ORR/PR/SD)
Nirogacestat	Pan-Notch	III	76% ORR in desmoid tumors
Brontictuzumab	Notch1	I/II	15% PR in NOTCH1-mutated tumors
CB-103	NICD/RBPJκ	I/II	40% SD in adenoid cystic carcinoma
AL101 + Pembrolizumab	Notch/PD-1	II	38% ORR in recurrent HNSCC
TAK-981	Notch	I/II	30% SD in solid tumors
RO6874054	Notch	I/II	25% PR in breast cancer

### 6.5 Future perspectives

The intricate crosstalk between the Notch signaling pathway and non-coding RNAs (ncRNAs) has not only reshaped our understanding of cancer and inflammatory pathogenesis but also opened unprecedented avenues for therapeutic innovation. As we advance, the integration of multidisciplinary tools—from single-cell transcriptomics to spatial omics and artificial intelligence—will be pivotal in unraveling the spatiotemporal dynamics of Notch-ncRNA networks. These approaches could map subtype-specific regulatory landscapes, particularly in tumors where Notch pathway dysregulation converges with ncRNA-driven epigenetic reprogramming, ultimately guiding the design of precision therapies tailored to individual molecular vulnerabilities.

Building on this foundation, overcoming current limitations in therapeutic specificity and delivery remains paramount. Emerging technologies such as nanotechnology and exosome engineering offer promising solutions by enabling targeted modulation of oncogenic ncRNAs (e.g., HOTAIR, MALAT1) or Notch receptors with minimal off-target effects. Simultaneously, combinatorial strategies that pair Notch inhibitors with immune checkpoint blockade or metabolic regulators may disrupt the plasticity of the tumor microenvironment, countering resistance mechanisms rooted in cellular adaptation. The synergy between CRISPR-based functional screening and machine learning could further accelerate the discovery of synthetic lethal nodes within Notch-ncRNA interaction networks, paving the way for novel multidrug regimens that exploit these vulnerabilities.

Translating these insights to the clinic requires bridging cutting-edge science with practical challenges. The development of non-invasive biomarkers—such as liquid biopsy panels tracking Notch-associated ncRNAs—and real-time imaging techniques to monitor pathway activity will revolutionize patient stratification and treatment monitoring. However, balancing therapeutic efficacy with tissue homeostasis demands innovative solutions: tissue-specific delivery systems (e.g., ligand-engineered nanoparticles) or transient epigenetic modulators may mitigate risks to normal stem cell niches while preserving antitumor activity. Beyond oncology, exploring the evolutionary conservation of Notch-ncRNA axes in chronic inflammation and autoimmune disorders could reveal shared pathogenic drivers, expanding therapeutic applications to a broader spectrum of diseases.

Ultimately, the convergence of mechanistic discovery and translational ingenuity promises to transform the Notch-ncRNA interplay from a complex biological paradigm into a cornerstone of clinical practice. By harmonizing molecular insights with advanced engineering and clinical validation, this field holds the potential to deliver durable, personalized therapies that transcend traditional boundaries between cancer and inflammatory diseases, offering new hope for patients worldwide.
